# Supplementary material for: Inflammatory factors and risk of lung adenocarcinoma: a Mendelian randomization study mediated by blood metabolites
Source: Front Endocrinol (Lausanne). 2024 Aug 27;15:1446863. doi: 10.3389/fendo.2024.1446863 (PMC11384989; doi:10.3389/fendo.2024.1446863)
Supplement: Supplementary file 1 [file DataSheet1.zip › supplementary files/S2/GCST90200097/sensitivity-analysis.pdf]

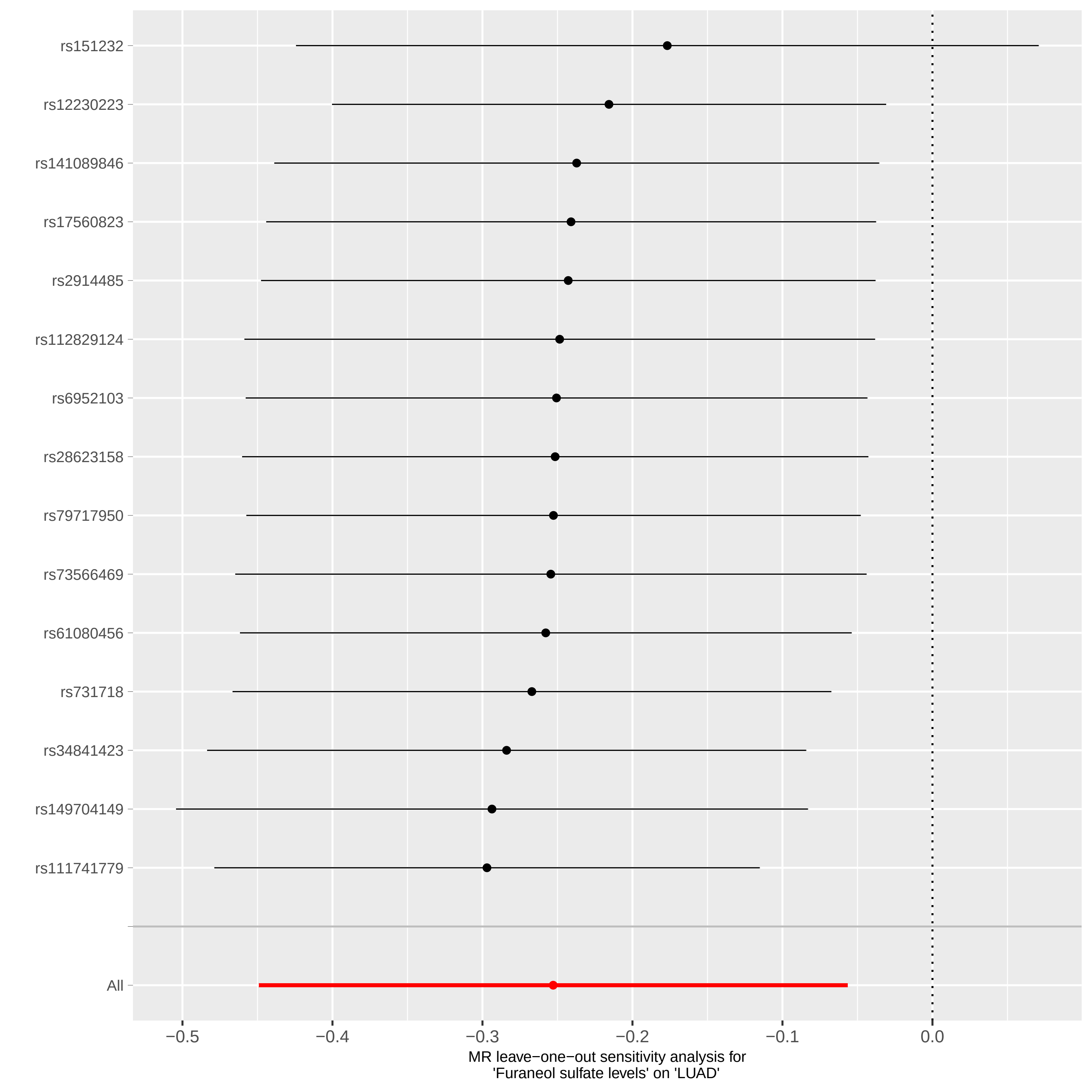

rs151232
rs12230223
rs141089846
rs17560823
rs2914485
rs112829124
rs6952103
rs28623158
rs79717950
rs73566469
rs61080456
rs731718
rs34841423
rs149704149
rs111741779
All
−0.5
−0.4
−0.3
−0.2
−0.1
0.0
MR leave−one−out sensitivity analysis for
'Furaneol sulfate levels' on 'LUAD'
